# A novel approach for efficient co-expression of two foreign genes based on the reverse genetic system of Newcastle disease virus

**DOI:** 10.3389/fmicb.2024.1442551

**Published:** 2024-12-18

**Authors:** Ting Lan, Qilong Liu, Jinying Ge, Yong Wang

**Affiliations:** ^1^Department of Epidemiology, School of Public Health, Harbin Medical University, Harbin, China; ^2^State Key Laboratory of Veterinary Biotechnology, Harbin Veterinary Research Institute, Chinese Academy of Agricultural Sciences, Harbin, China

**Keywords:** Newcastle disease virus, reverse genetic system, recombinant virus, foreign gene, co-expression

## Abstract

Newcastle disease virus (NDV) is an ideal model for exploring the mechanisms of the virus; it is also an optimal vector for developing vector vaccines and for cancer therapy. A reverse genetic system of NDV Mukteswar strain controlled by eukaryotic cellular RNA polymerase II promoter was established by reverse genetics technology. Based on the reverse genetic system, an open reading frame of the enhanced green fluorescent protein (EGFP) gene be inserted between the P and M genes of the viral genome and flanked with the gene start (GS) sequence and gene end (GE) sequence to form an independent transcription unit. The rescued virus was amplified in specific pathogen-free (SPF) chicken embryos for 10 generations, and the results showed that the recombinant virus could stably express the exogenous gene for at least 10 generations. Efficient expression of two exogenous genes synchronously is essential for the development of NDV-based multivalent vaccine candidates. Explore the possibility of simultaneous and efficient expression of two exogenous genes based on NDV vector. In the present study, a recombinant virus with co-expression of EGFP and cherry fluorescent protein (CFP) inserted between the intergenic regions of the P/M gene as two independent transcription units was successfully rescued. The results showed that the two exogenous genes could be expressed synchronously and efficiently. The results of biological analysis of the expression efficiency of exogenous genes showed that the EGFP in recombinant viruses with two exogenous genes was slightly lower than that of recombinant viruses with one exogenous gene, but the expression efficiency of CFP in recombinant viruses with two exogenous genes was higher than EGFP in both viruses. These recombinant viruses have similar growth kinetics but with a little attenuation in virulence compared with parental viruses. In conclusion, these data indicated that this study successfully established the reverse genetic system of the NDV Mukteswar strain and achieved the purpose of efficient expression of two exogenous genes synchronously in a novel approach, laying the foundation for the development of multivalent vaccines or tumor therapeutics using NDV as a vector.

## 1 Introduction

Newcastle disease (ND) is a highly contagious infectious disease of avian species with worldwide distribution which has an important economic impact on the poultry industry. The causative agent, Newcastle disease virus (NDV), is a member of the genus Avulavirus in the family Paramyxoviridae (King et al., 2012). Almost all species of birds are susceptible to NDV. Depending on the severity of clinical symptoms, NDV isolates can be classified into three pathotypes: (1) lentogenic (non-virulent), such as LaSota and BI, which cause inapparent disease; (2) mesogenic (intermediate), such as Beaudettc C and Mukteswar, which could cause intermediate respiratory symptoms; and (3) velogenic (highly virulent), such as outbreak isolates, which cause very serious disease, the mortality could be up to 100%. Although NDV isolates can be classified into a variety of genotypes, all of them have only one serotype. Naturally occurring lentogenic strains (e.g., LaSota) are widely used as live vaccines to prevent and control the disease in poultry industry. Globally, vaccination is the preferred means of controlling ND. Some isolated mesogenic strains have been used as a booster vaccine in endemic countries; some of these strains have inherent oncolytic activity and can selectively replicate in tumor cells but not in normal human cells. The safety record of more than 60 years of NDV vaccine use has shown that the virus is safe for mammals and primates, sometimes exposure to diseased birds may cause infection in human beings, but no data showed that the virus could transmitted from person to person (Alexander, 1989; Beard and Hanson, 1984).

The genome of NDV is a non-segmented, single-strand, negative-sense RNA (Alexander, 1989). The length of the genome is approximately 15 kb, which consists of six independent transcription units (ITUs) flanked with 3′leader and 5′trailer. From the 3′ to 5′ terminal, these six ITU encode six structural proteins: nucleoprotein (*NP*), phosphoprotein (*P*), matrix protein (*M*), fusion protein (*F*), hemagglutinin-neuraminidase protein (*HN*), and large protein (*L*) (Millar et al., 1988). Each gene open reading frame (ORF) is flanked with gene start (GS), gene junction (GJ), and gene end (GE) sequences. Through an editing mechanism, the messenger RNA of the phosphoprotein gene could express two additional proteins: V and W, which influence the characteristics of NDV (Steward et al., 1993). To date, study results showed that the amino acids sequence of the F protein cleavage site is a major determinant of virulence (McGinnes et al., 2002; Ogasawara et al., 1992; Peeters et al., 1999). The genome of NDV is a negative-sense RNA, so the naked genomic RNA cannot replication; it must be formed the ribonucleoprotein complex together with NP, P, and L proteins, which served as a template for RNA transcription and virus replication (Lamb and Kolakofsky, 2001).

Schnell first described rescued recombinant rabies virus from a full-length cDNA clone based on reverse genetic technology in 1994 (Schnell et al., 1994). Since then, the reverse genetic system of a variety of non-segmental RNA viruses has been subsequently completed (Conzelmann, 1996; Durbin et al., 1997; Garcin et al., 1995; Hasan et al., 1997; He et al., 1997; Lawson et al., 1995; Radecke et al., 1995; Baron and Barrett, 1997; Clarke et al., 2000; Volchkov et al., 2001). The reverse genetics system of NDV was first reported in 1999, two independent research groups successfully rescued recombinant NDV, lentogenic strain LaSota, from full-length cDNA clone almost simultaneously (Peeters et al., 1999; Römer-Oberdörfer et al., 1999). Since then, all three pathotypes of NDV strains have been rescued from full-length genome cDNA clones and explored as vectors to express various foreign genes for vaccine or gene therapy purposes (Peeters et al., 1999; Römer-Oberdörfer et al., 1999; Krishnamurthy et al., 2000; Paldurai et al., 2014; Li et al., 2011; Xiao et al., 2012; Liu et al., 2007; Wang et al., 2015; Sun et al., 2020; Huang et al., 2004). In addition, Ge et al. (2007) rescued a recombinant NDV, LaSota strain, which expresses the hemagglutinin of H5 subtype highly pathogenic avian influenza virus (HPAIV), and developed a commercial bivalent vaccine for the control of the H5 subtype HPAI and ND. The established reverse genetic system of NDV made it possible to manipulate the viral genome to research the function of genes and other characteristics of the virus; it is also a facility platform to develop more safe, stable, and efficient vaccines or as a vector to develop bivalent/multivalent vaccines. Conventionally, the reverse genetic systems of a non-segmented, single-strand, negative-sense RNA virus were controlled by the T7 polymerase promoter, which needs an additional T7 RNA polymerase provided either by a recombinant vaccinia virus (Fuerst et al., 1986) or a stable cell line which could constitutively express T7 RNA polymerase (Buchholz et al., 1999). The reverse genetic system is involved in the co-transfection of the full-length cDNA plasmid with helper plasmids that express the *NP*, *P*, and *L* proteins to rescue the infectious virus. Based on the reverse genetic techniques, various non-segmented negative-strand RNA viruses were recovered from full-length cDNA clones (Gabriele Neumann et al., 2002; Conzelmann, 2004).

To improve the rescue efficacy and simplify the procedure of non-segmented negative-strand RNA viruses’ reverse genetic system, some researchers developed a eukaryotic promoter-based reverse genetic system for various RNA viruses, which avoided the additional T7 RNA polymerase and improved the recovery efficiency (Fodor et al., 1999; Kenichi et al., 2003; Tayeb et al., 2015; Martin et al., 2006). To generate precise RNA ends, Le Mercier et al. (2002) added the hammerhead ribozyme (HamRz) and hepatitis delta virus ribozyme (HdvRz) sequences at the leader and trailer of the genome cDNA, respectively. Here, we report the reverse genetic system of the NDV Mukteswar strain, the full-length genome cDNA flanked with HamRz and HdvRz sequences at the 5′ end and 3′ end of the antigenome. The full-length genome cDNA plasmid and three helper plasmids were all controlled by the CMV promoter. A foreign gene, an enhanced green fluorescent protein (EGFP) gene as a reporter, was inserted into the genome and rescued the recombinant virus, and the results were confirmed by the presence of green fluorescence in infected cells. Studies have shown that the non-coding region between the phosphoprotein (P) gene and matrix protein (M) gene in the NDV genome is the optimal site for foreign gene insertion and expression (Zhao and Peeters, 2003; Carnero et al., 2009; Zhao et al., 2015). The expression level of foreign genes in recombinant NDV is related both to its length and its location in the genome, which is more important. To date, most of the recombinant NDVs generated express one or two exogenous genes that are inserted as separate transcriptional units into different intergenic regions; the expressing level of foreign genes was varied because the gene transcription of the NDV gradient decreased across the order of genes, and from 3′ leader to 5′ trailer (Kristina Ramp et al., 2011; DiNapoli et al., 2010; Takaaki Nakaya et al., 2001; Lamb and Parks, 2013; Samal, 2011). Some researchers use the internal ribosomal entry site (IRES) sequence or the viral 2A self-cleavage peptides combined with independent transcription unit-generated recombinant NDV to co-express two foreign genes. But regardless of the use of IRES or viral-2A self-cleavage peptides methods, the expressing level of foreign gene is lower than the independent transcription unit approach. Another concern about the viral 2A self-cleavage peptides is that the extra short 2A peptide residues may alter the biological functions or immunogenicity of these foreign proteins, potentially affecting the efficacy of the vaccine (Zhang et al., 2015; Hu et al., 2017; Xu et al., 2018; Hu et al., 2018; He et al., 2020; Wen et al., 2015). Although there are many factors influencing the protective immunity induced by vector vaccines, the expression level of exogenous genes is undoubtedly the most important factor.

In the present study, we constructed a reverse genetic system based on the backbone of the NDV Mukteswar strain and rescued the recombinant virus from a full-length cDNA clone plasmid. The full-length genome cDNA was placed between the HamRz and HdvRz sequences to create a precise genome terminal. The transcription of the full-length cDNA plasmid and three helper plasmids was all controlled by the CMV promoter. On the other hand, the EGFP gene as a reporter is inserted between the non-coding regions of the P and M genes and rescued the recombinant virus, the results confirmed by the presence of green fluorescence in infected cells. To obtain a high level of expression and minimize the difference in the expression level of two foreign genes, we generated a Mukteswar strain-based recombinant NDV that co-expressing two foreign genes, EGFP and CFP genes, that is linked with gene end(GE)-gene junction(GJ)-gene start(GS) sequence motif. The gene junction has only one nucleotide. The results showed that the GE-GJ-GS motif as a linker is an effective method for the simultaneous and efficient expression of two exogenous genes by recombinant NDV at the optimal insertion sites of the genome. The data obtained from the study indicated that the new method has the potential to develop NDV-based multivalent vector vaccines, improve the immune efficacy of bivalent vector vaccines, or enhance the oncolytic properties of recombinant NDV for tumor therapy.

## 2 Materials and methods

### 2.1 Cells and viruses

HEp-2 cells were grown in Dulbecco’s Modified Eagle Medium (DMEM, Thermo Fisher Scientific, Carlsbad, CA, United States) containing 10% fetal bovine serum, maintained at 37°C, and 5% CO2, supplemented with antibiotics (100 U/mL of penicillin and 100 mg/mL of streptomycin). NDV Mukteswar strain (provided by Harbin Weike Biotechnology Development Company, Harbin City, Heilongjiang Province, China) was injected into embryonated 10-day-old specific pathogen-free (SPF) chicken eggs to amplify the virus. The allantoic fluid of SPF chicken eggs infected with the Mukteswar strain was collected 3-day post-inoculation, aliquot, and frozen in a refrigerator at −80°C.

### 2.2 Construction of the full-length cDNA clone of NDV from RT-PCR products

Viral RNA was extracted from the allantoic fluid samples infected with the NDV Mukteswar strain using the QIAGEN viral RNA mini kit following the manufacturer’s instructions. Reverse transcription was carried out with HiScript®II one-step RT-PCR Kit (Vazyme Corp., China) using specific primers that were complementary to the viral genome (Table 1) to generate fragmented cDNAs, which were then used to construct the full-length cDNA clone. The whole viral genome is divided into four fragments, and each fragment has an overlapping region of approximately 20 nt with its adjacent fragments (Table 1).

**Table 1:** Primers used in cDNA amplification of the NDV Mukteswar strain.

Muk-1F	5’-GGAAAGGAATTCCTATAGTCACCAAACAGAGAATCCGTGAG-3’
Muk-1R	5’-CTTGTATCAGAGCC**GCGGCCGC**CGTTATC-3’
Muk-2F	5’-GATAACG**GCGGCCGC**GGCTCTGATACAAG-3’
Muk-2R	5’-GTCACTAATTTGTTGACTGC**AGATCT**TGTTC-3’
Muk-3F	5’-AACA**AGATCT**GCAGTCAACAAATTAGTGAC-3’
Muk-3R	5’-GATCAACACGGATGATGCCCTTAG-3’
Muk-4F	5’-AAGGGCATCATCCGTGTTGATCTG-3’
Muk-4R	5’-GAGGCTGGGACCATGCCGGCCACCAAACAAAGATTTGGTG-3’

^aSequences complementary to ribozymes are underlined. bArtificially mutated restriction enzyme sites are indicated in bold.^

To distinguish with the wild-type Mukteswar strain, two restriction enzyme sites were introduced into the full-length cDNA clone. The *NotI* site was introduced into the genome at nucleotide 4,954 as a genetic marker to facilitate the introduction of a restriction enzyme site, *PmeI,* which was used in inserting foreign genes. Another genetic marker, *BglII*, was introduced at position 8,957. The two restriction sites were introduced into the genome by using specific synonymous mutation primers. These four segments each of them has an overlapping region of approximately 20 nt with each neighbor at both sides, coding the complete genome sequence of the Mukteswar strain, to be cloned into an intermediate vector, pBluescript II K/S (+/−). All four plasmids were sequenced to confirm that the sequence were consistent with the genome and then used as templates to amplify these four fragments using high-fidelity DNA polymerase-2 × Phanta® Max Master Mix (Vazyme Corp., China) and purified with FastPura® Gel DNA Extraction Mini Kit (Vazyme). Based on the specific primers, there is an overlapping region of 20 nt homology with HamRz at the head of the first fragment, and at the trailer of the fourth fragment there is an overlapping region of 21 nt homologous to HdvRz (Table 1).

The full-length genome cDNA be cloned into a eukaryotic expression vector, namely, the pCI plasmid (Promega), flanked with HamRz and HdvRz sequence. To generate a linearized vector containing HamRz sequence and HdvRz sequence, using a multi-step fusion PCR protocol to make the HamRz just following the CMV promoter. The same protocol has been used to introduce the HdvRz sequence upstream of the SV40 Late polyA sequence. The result is that the linearized vector has the HamRz sequence and HdvRz sequence, designed as pCI-HamRz/HdvRz.

The linearized vector and these four cDNA fragments of NDV genome were connected by a multi-fragment ligation kit, CloneExpress® Ultra One Step Cloning Kit (Vazyme Corp., China), following the manufacturer’s instructions to generate the full-length genome cDNA clone of Mukteswar strain. Conduct the transformation with Stb12 competent cells following the user guidelines, all steps are performed at 30°C. The full-length genome cDNA plasmid was designated as p-N/Muk.

### 2.3 Construction of rMuk-based cDNA clones containing the EGFP gene and EGFP/CFP genes, respectively

The full-length genome cDNA clone plasmid, p-N/Muk, is used as a template to construct the recombinant plasmid through the introduction of an additional restriction enzyme site, *PmeI,* in the non-coding intergenic region of P and M genes. Four pairs of specific primers that have an overlapping region of homology approximately 20 nt with each neighbor were designed to generate this full-length plasmid, as mentioned above. PCR was carried out using the plasmid p-N/Muk as a template to amplify these four fragments with the high-fidelity DNA polymerase-2 × Phanta® Max Master Mix (Vazyme Corp., China). Then utilizing CloneExpress® Ultra One Step Cloning Kit (Vazyme Corp., China), following the manufacturer’s instructions to generate the mutated full-length cDNA clone plasmid, named p-N/Muk (*PmeI*), as described above.

A pair of specific primers were designed to amplify the EGFP gene of the plasmid pIRES2-EGFP. The up primer containing the GE-GJ-GS motif, in the upstream of the motif is a *PmeI* restriction enzyme site, and the downstream primer also have a *PmeI* site at the 3′ end. As described above, at the 5′end of up primer and down primer, there is a homologous overlapping region of approximately 20 nt with the genome, respectively. To generate a linearized full-length cDNA vector, digested the plasmid p-N/Muk (*PmeI*) with the restriction enzyme, *PmeI*. Then use the ligation reaction kit, ClonExpress® II One Step Cloning Kit (Vazyme Corp., China), following the manufacturer’s directions to generate the recombinant full-length cDNA plasmid, designed as p-N/Muk-EGFP.

Similar to the one described above, we use a pair of specific primers to amplify the CFP gene of plasmid p-N-cherry, at the head of the PCR product, which has a 21 nt sequence containing the GE-GJ-GS motif. Another pair of specific primers to amplify the EGFP gene which has an overlapping region of homology approximately 20 nt with the leader of CFP at the trailer of the EGFP gene. Then the linearized vector, amplified EGFP fragment and amplified CFP fragment were put together to make the ligation reaction with the CloneExpress® Ultra One Step Cloning Kit (Vazyme Corp., China), following the manufacturer’s instructions to generate the recombinant full-length cDNA clone plasmid, designed as p-N/Muk-E/C.

### 2.4 Construction of three helper plasmids

DNA fragments containing the open reading frames of the *NP*, *P,* and *L* genes were amplified from the full-length genome cDNA clone plasmid by the high-fidelity DNA polymerase-2 × Phanta® Max Master Mix (Vazyme Corp., China) and cloned into the vector pCI, designed as pCI-NP, pCI-P, and pCI-L, respectively.

The *NP* and *P* cDNA were cloned into the pCI vector by using the *NheI* and *NotI* restriction sites, whereas the *L* cDNA was inserted into the pCI vector by using the *XbaI* and *NotI* sites.

### 2.5 Transfection and recovery of recombinant viruses

HEp-2 cells were grown overnight in six-well plates to 60–80% confluency in DMEM medium supplemented with 10% FBS. The cells were washed twice with DMEM medium prior to operate and replaced with Opti-MEM (Invitrogen Corp., Carlsbad, CA, United States) at a volume of 2 mL per well. Co-transfection was then conducted using p-N/Muk, p-N-Muk/EGFP, and p-N-Muk-E/C with three help plasmids: pCI-NP, pCI-P, and pCI-L, respectively. Each well contains a total of 5.0 μg of the plasmids at a ratio of 4:2:2:1 diluted in ExFect® Transfection Reagent (Vazyme Corp., China), following the manufacturer’s recommendations. At 6–8 h post-transfection, the medium was replaced with DMEM supplemented with 5% FBS. After 3–5 day post-transfection, the culture medium and cell monolayers were harvested (depending on the cell pathogenicity effect, CPE), and then the cell suspension was inoculated into an allantoic cavity of 10-day-old embryonated SPF eggs. Three-day post-inoculation, the allantoic fluid was harvested and viruses were detected with the hemagglutinating activity (HA) test using 1% chicken erythrocyte in PBS (Alexander, 1998). Recombinant viruses carrying reporter genes can be observed with green fluorescent and green fluorescent/cherry fluorescent, respectively, by inverted fluorescence microscopy. Viral RNA was extracted from the rescued virus and confirmed by RT-PCR. The fragments that harbored the mutated genetic markers were then detected by sequencing and digested the RT-PCR products with *NotI* and *BglII*. These rescued viruses were amplified in SPF chicken embryos and harvested, aliquoted, and stored at −80°C as a stock, and be named rMuk, rMuk-EGFP, and rMuk-E/C, respectively.

### 2.6 Biological characteristics of the rescued rMuk, rMuk-EGFP, and rMuk-E/C

To evaluate the genetic stability of the rescued recombinant viruses, we successively passaged the recombinant virus, rMuk, in embryonated 10-day-old SPF chicken eggs 10 times. The 5th and 10th generation virus, viral RNA was extracted from the harvested allantoic fluid, and RT-PCR analysis, *NotI,* and *BglII* digestion were performed to determine the presence of the genetic markers.

The standard mean death time (MDT) assay was conducted to assess the pathogenicity and virulence of these recombinant viruses in embryonated 10-day-old SPF chicken eggs following the standard procedure of the OIE manual (Alexander, 1989; Office International des Epizooties, 2004). Titers of these recombinant viruses were tested following the standard protocol of HA test, the 50% tissue culture infective dose (TCID50) and the 50% egg infective dose (EID50) assay in 10-day-old SPF chicken eggs (Alexander, 1989), as described previously (Ge et al., 2007).

To investigate the effect of foreign gene insertion on the growth of recombinant viruses, these rescued recombinant viruses, rMuk, rMuk-EGFP, and rMuk-E/C, were inoculated with BHK-21 cells in 96-well plates at an MOI, 0.01, and incubated at 37°C in 5% CO_2_. Samples are collected at intervals of 8h, 3 samples per time point, up to 96 h. Viral titers were determined by an EID_50_ titration in 10-day-old SPF chicken eggs for each time point in triplicates, as described previously (Ge et al., 2007).

### 2.7 Examination and comparison of EGFP and CFP fluorescence intensities

Referring to YU’s method (He et al., 2020), HEp-2 cells monolayer grown in 96-well plates were infected with recombinant viruses, rMuk-E/C and rMuk-EGFP at MOI 0.01, respectively, and incubated at 37°C in 5%CO_2_. The expression levels of EGFP and CFP were quantitated by measuring the fluorescence intensity with a Fluorescence Microplate reader every 24 h post-infection. The Microplate Reader with a 485/20 excitation filters and 528/20 emission filters for EGFP, and 587/35 excitation filters and 610/40 emission filters for CFP. For quantitative comparison, three replicates were set up for each sample, and the fluorescence intensity was represented as the average of the three samples. Fluorescence intensity carves of EGFP or CFP expressed by the two recombinant viruses at different points were plotted. Compare and analyze the intensity difference between fluorescence of double and single exogenous gene expressions of recombinant viruses using a *t*-test with 5% significance (Microsoft Excel).

## 3 Results

### 3.1 Construction of the full-length genome cDNA clone of the Mukteswar strain and recombinant full-length plasmids containing the EGFP and EGFP/CFP, respectively

**Figure 1:**
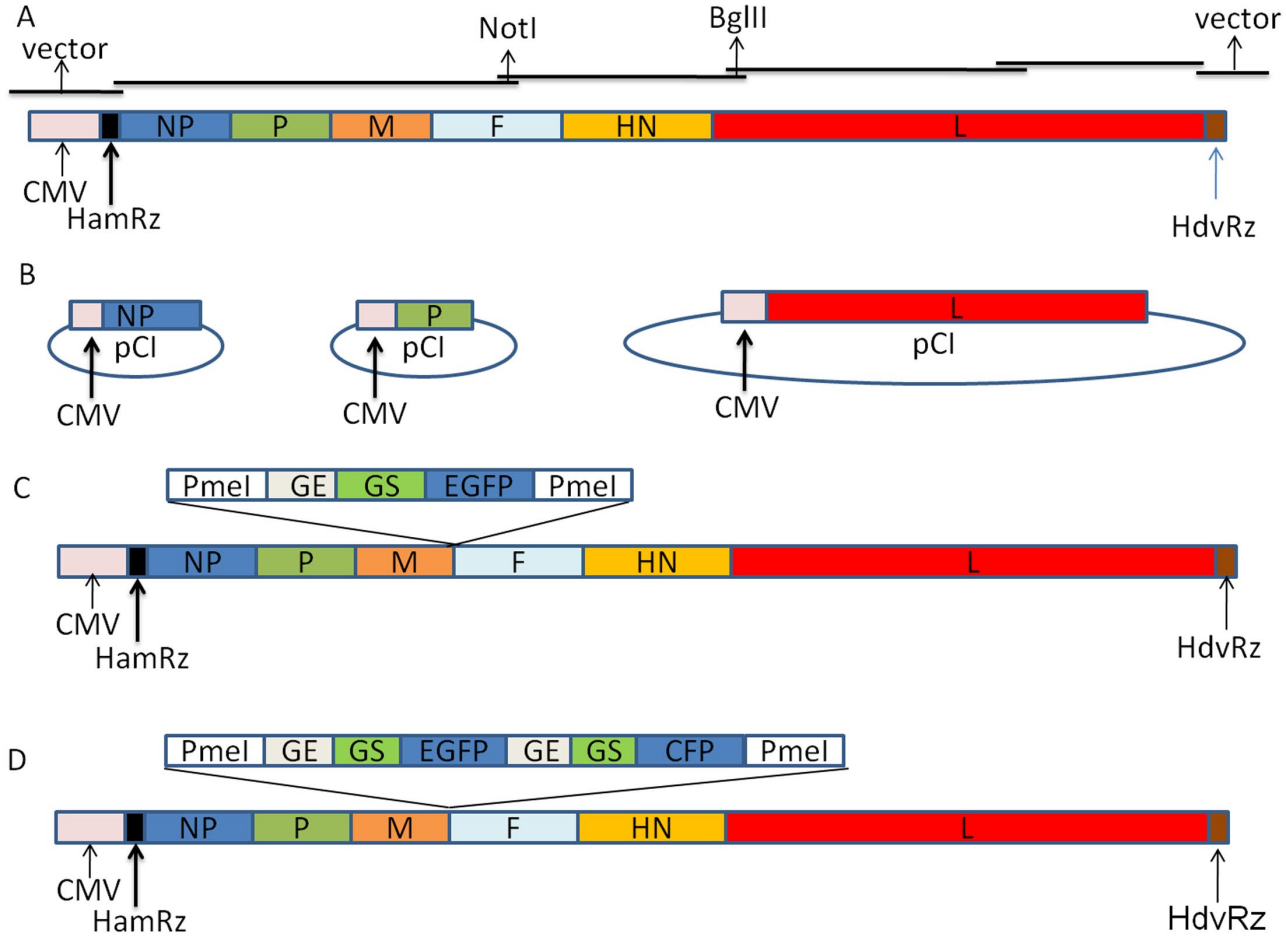
Schematic representation of NDV Mukteswar strain-based expression vector. **(A)** Cloning strategies for constructing full-length cDNA from four sub-genomic fragments show that the full-length cDNA construction of Mukteswar strains was promoted by the CMV promoter. **(B)** Three help plasmids. The ORF of NP, P, and L gene were inserted into vector pCI, respectively. **(C)** An expanded view of the EGFP-expressing recombinant Mukteswar strain is shown. ‘HamRz’ and HdvRz’ refer to the hammerhead ribozyme and hepatitis delta virus ribozyme sequences, respectively. **(D)** The GE-GJ-GS motif was used as an adapter to link two foreign genes as two independent transcription units. Since GJ has only one nucleotide, it is simplified to GE-GS in the figure.

To construct the full-length genome cDNA clone of the Mukteswar strain, the complete genome of the virus is assembled from four overlapping fragments using the CloneExpress® Ultra One Step Cloning Kit (Vazyme Corp., China) connected with the linearized vector, pCI-HamRz/HdvRz. The result is that the 5′ and 3′ ends of the antigenome of NDV were precisely linked to HamRz and HdvRz, respectively. The transcription of the full-length cDNA is controlled by the CMV promoter and designated as p-N/Muk (Figure 1A). Two genetic tags were introduced into the genome to distinguish the recombinant virus from the wild-type virus. The open reading frames of the *NP*, *P*, and *L* genes were cloned into vector pCI and designated as pCI-NP, pCI-P, and pCI-L (Figure 1B). To develop the virus as a vector for expressing foreign genes, the EGFP gene and EGFP/CFP genes were inserted into the intergenic region of P/M genes and were designated as p-N/Muk-EGFP and p-N/Muk-E/C, respectively (Figures 1C,D). The length of the three full-length genome cDNA plasmids obeys “the rule of six” (Kenichi et al., 2003). The HamRz and HdvRz sequence motifs could produce the exact 5′ end and 3′ end of the antigenome RNA.

#### 3.1.1 Generation of infectious recombinant NDV from full-length cDNA clone

The full-length genome cDNA of the NDV Mukteswar strain was assembled in plasmid pCI and designated as p-N/Muk. Based on this plasmid, two other full-length plasmids that were inserted with EGFP or EGFP/CFP in the intergenic region of P/M were constructed and designated as p-N/Muk-EGFP and p-N/Muk-E/C, respectively. To rescue these recombinant NDVs, HEp-2 cells were grown overnight in a six-well plate to 60–80% confluency in DMEM supplemented with 10% FBS. Co-transfection experiments using ExFect® Transfection Reagent (Vazyme Corp., China) were performed, following the manufacturer’s instructions. Details of the system are presented in Figure 1. Three helper plasmids express three proteins that are essential for viral replication. These three proteins together with the viral genomic RNA, constituted the ribonucleoprotein (RNP) complex that initiates the life cycle of NDV. The supernatant and cell monolayers were harvested 3–5 days post-transfection and injected into the allantoic cavities of 10-day-old SPF embryos to amplify the rescued virus. Three days later, some embryos were found dead and were transferred into a 4°C freezer for several hours, then tested for hemagglutinating activity of the allantoic fluid. Any HA-positive allantoic fluid samples were collected and designated as harboring the rescued recombinant virus.

**Figure 2:**
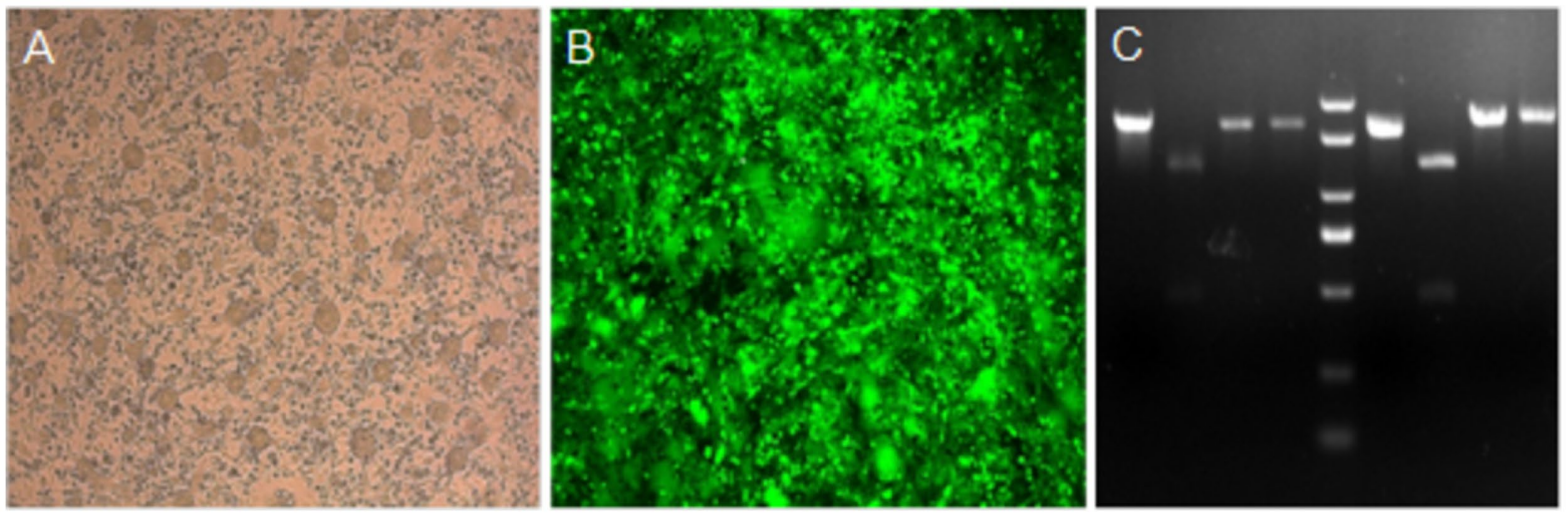
Rescued recombinant viruses infected HEp-2 cells and restriction enzyme digestion of RT-PCR products. **(A)** HEp-2 cells were infected with rMuk at an MOI of 0.1. **(B)** HEp-2 cells were infected with rMuk-EGFP at an MOI of 0.1. EGFP expression was observed at 24 hpi. **(C)** Total RNA was prepared from HA-positive allantoic fluid of rMuk and analyzed by RT-PCR and restriction enzyme digestion. Lane 5 shows the molecular weight marker.

**Figure 3:**
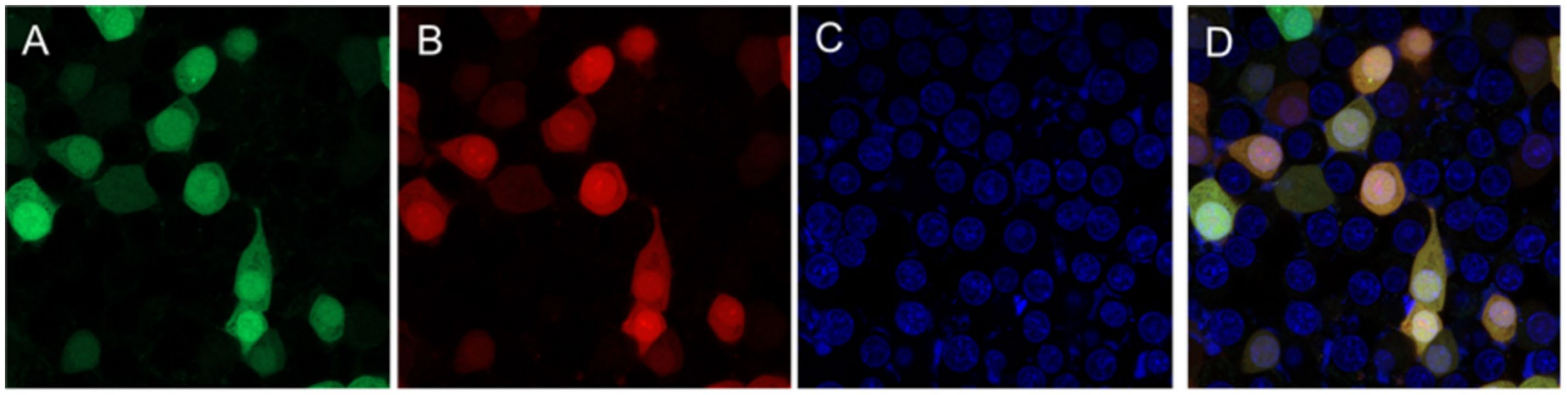
Two fluorescence expressions of the rMuk-E/C virus were detected by confocal microscopy. HEp-2 cells were infected with rMuk-E/C virus at an MOI 0.01, 24 hpi, and infected cells were checked for the corresponding fluorescence from the same field and digitally photographed at 100 × magnifications. **(A)** Green fluorescence. **(B)** Cherry fluorescence. **(C)** DAPI. **(D)** The merged EGFP and CFP **(A,B)** images.

These viruses rescued from plasmids p-N/Muk, p-N/Muk-EGFP, and p-N/Muk-E/C were designated as rMuk, rMuk-EGFP, and rM-E/C, respectively. The bright green fluorescence (Figure 2B) and green fluorescence/cherry fluorescence (Figure 3) were detected at 24 h post-infection (hpi) via an inverted fluorescence microscope and confocal microscopy.

These three recombinant viruses were passed 10 times in SPF embryos to test the genetic markers and inserted foreign genes were stably maintained in the genome. Detection of the genetic marker was conducted by RT-PCR, and the PCR products were digested with *NotI* and *BglII*, respectively (Figure 2C). Green fluorescence detection was conducted to verify that the EGFP gene was stably expressed in rMuk-EGFP-infected cells. Green fluorescence and cherry fluorescence detection were conducted to verify that the EGFP gene and CFP gene were stably expressed in rMuk-E/C-infected cells. The results of RT-PCR sequencing of these recombinant viruses also showed that foreign genes and genetic tags could be stable and maintained for at least 10 generations (the results were not shown).

#### 3.1.2 Biological characteristics of recombinant viruses

The biological characteristics of generated recombinant viruses were compared by the standard procedure as described in the OIE manual (Office International des Epizooties, 2004) to determine the MDT in 10-day-old embryonated SPF chicken eggs. These MDT results were tested by *t*-test, showing that there was no significant difference (*p* > 0.05) in MDT between the three recombinant viruses (Table 2).

**Table 2:** Biological properties of these rescued recombinant viruses.

	Passages	MDT^a^	EID_50_^b^
rMuk	F3	64	8.0
rM-EGFP	F3	70	8.0
rM-E/C	F3	75	8.17

^aMDT: Mean death time assay in embryonated SPF eggs. bEID50: 50% egg infective dose assay in embryonated SPF eggs.^

**Figure 4:**
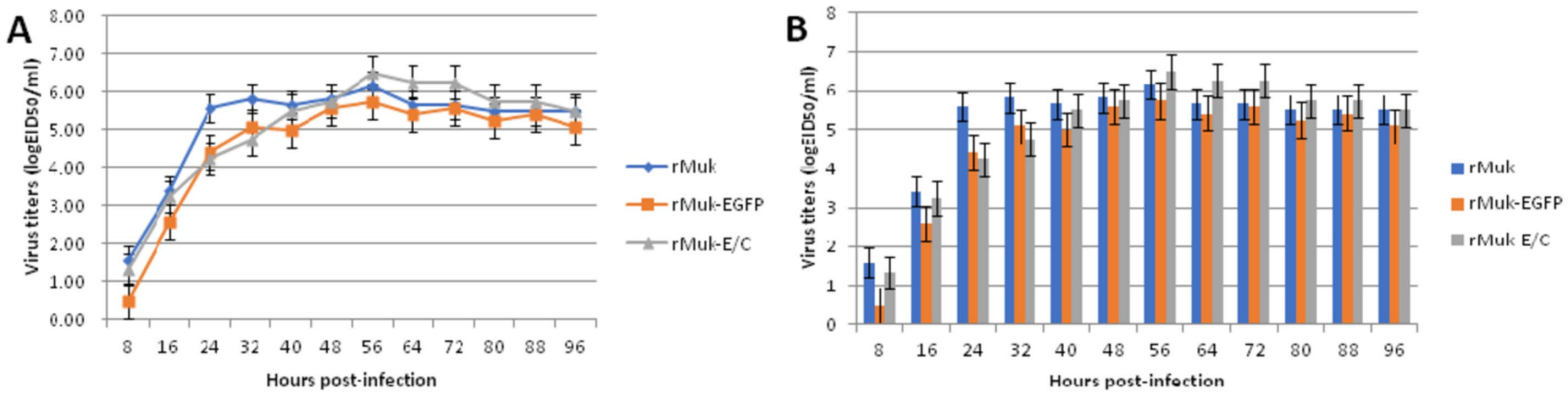
Multistep growth curves of the rMuk, rMuk-EGFP, and rMuk-E/C. BHK-21 cells were infected with studied viruses at an MOI of 0.01, respectively. Viral titers in infected cell supernatants were determined at the time points of 8, 16 24, 32, 40, 48, 56, 64, 72, 80, 88, and 96 h post-inoculation. Data shown were acquired from three repeats. **(A)** Multistep growth kinetics are presented as line charts. **(B)** Multistep growth kinetics are presented as histograms.

HEp-2 cells were infected with the recombinant virus, rMuk-E/C, at 0.01 MOI. The co-expression of EGFP and CFP at 24 h post-infection was observed by inverted fluorescence microscopy and confocal microscopy, as shown in Figure 3. The expression of EGFP and CFP can be observed. Syncytia caused by NDV can be seen under the light microscope. Under the fluorescence microscope, EGFP and CFP expressions can be observed over a large area, and the fluorescence intensity is strongest in syncytia. After merging the fluorescent images, EGFP and CFP are co-expressed in every virus-infected cell. The growth kinetics showed that the replication of the recombinant viruses was only slightly delayed in the early stages of infection (first 36 h), after which the two recombinant viruses could replicate to similar titers compared to the parent strain, rMuk strain (Figure 4).

#### 3.1.3 Comparison of exogenous gene expression levels

**Figure 5:**
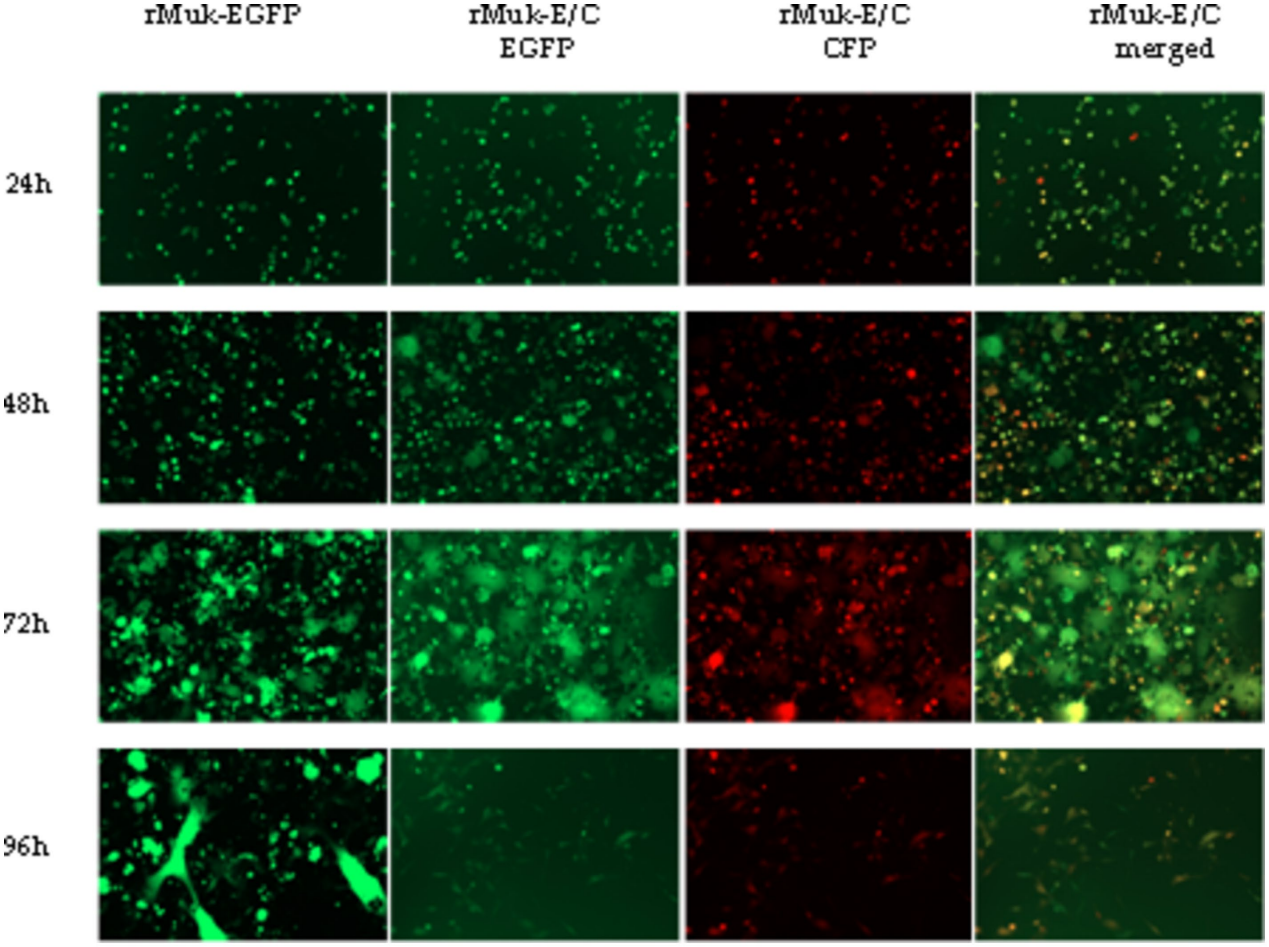
EGFP and CFP expressions in recombinant virus-infected cells were detected by fluorescence microscopy. HEp-2 cells were infected with the indicated recombinant viruses at 0.01 MOI. Every 24 h post-infection, infected cells were examined under an inverted fluorescence microscope. EGFP and CFP fluorescence from the same field of the infected cells was digitally photographed. Fluorescence intensity is determined using a Fluorescence Microplate Reader.

**Figure 6:**
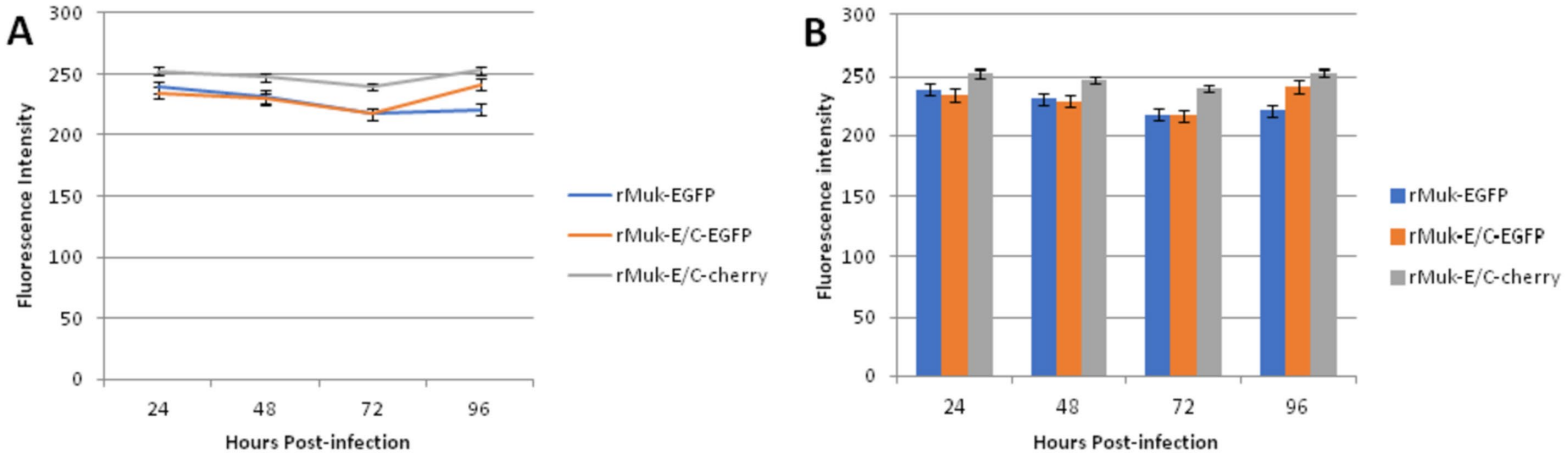
Comparisons of the difference between EGFP and CFP expressed from recombinant virus-infected HEp-2 cells. HEp-2 cells were grown in 96-well plates and infected with the recombinant NDV, rMuk-EGFP, and rMuk-E/C at 0.01 MOI. Every 24 h post-infection, EGFP and CFP fluorescence intensities were measured via fluorescence microplate reader in triplicate wells. **(A)** The mean fluorescence intensities of EGFP and CFP at different time points after infection were plotted. Error bars indicate the standard deviation (SD) of EGFP or CFP fluorescence intensity. **(B)** The mean fluorescence intensities of EGFP and CFP at different time points after infection are plotted as histograms. Error bars indicate the standard deviation (SD) of EGFP or CFP fluorescence intensity.

The fluorescence intensities of EGFP and CFP in virus-infected HEp-2 cells were determined to compare the expression efficiency of the two recombinant viruses. As shown in Figure 5, EGFP and CFP fluorescence intensities reach very high levels 24 h post-infection; afterward, fluorescence intensity decreases slowly, reaching its lowest value 72 h post-infection, followed by a rise slowly. The key points that attract attention are: there was no difference in the intensity of EGFP expression between rMuk-E/C and rMuk-EGFP; the *t*-test results showed that there was no difference in fluorescence intensity between the two virus-infected cells (*p* > 0.05). However, CFP, as the second foreign gene, had higher fluorescence intensity in infected cells than EGFP and also higher than EGFP expressed in rMuk-EGFP-infected cells. The fluorescence intensity of CFP was significantly different from EGFP that was expressed by rMuk-EGFP or rMuk-E/C (*p* < 0.05), as indicated in the histogram of fluorescence intensity. These results indicate that when two foreign genes are expressed simultaneously in the NDV genome, using the GE-GJ-GS motif as an adapted linker is an effective method for the simultaneous expression of two exogenous genes at the optimal insertion site. The efficient expression of two exogenous genes can be achieved simultaneously, and the expression level of two exogenous genes seemed better than that of a single exogenous gene (Figure 6).

## 4 Discussion

Since the successful establishment of reverse genetic technology in 1994 (Schnell M. J. et al., 1994), a large number of single-strand, negative-sense RNA viruses have been successfully rescued from full-length genome cDNA clones via different strategies to express various foreign proteins or to improve the understanding of the virus. Peeters et al. (1999) and Römer-Oberdörfer et al. (1999) rescued the lentogenic NDV, respectively, almost at the same time. Since then, several groups have generated recombinant NDV from full-length genome cDNA clones, including lentogenic, mesogenic, and velogenic strains (Römer-Oberdörfer et al., 1999; Krishnamurthy et al., 2000; Paldurai et al., 2014; Li et al., 2011; Xiao et al., 2012; Liu et al., 2007; Wang et al., 2015; Sun et al., 2020; Huang et al., 2004). In recent decades, reverse genetic technology has served as a powerful tool applied in studying the mechanisms of pathogenesis and exploring the biological functions of viral proteins; it is also an ideal system for the development of live attenuated or engineered vector vaccines (Conzelmann, 1996; Paldurai et al., 2014; Xiao et al., 2012; Wang et al., 2015; Sun et al., 2020; Huang et al., 2004; Ge et al., 2007; Neumann et al., 2002; Zhang et al., 2015).

In the present study, we generated a recombinant mesogenic NDV, the Mukteswar strain, from full-length cloned cDNA. In the system, we utilize a eukaryotic cell RNA polymerase system to replace the conventional T7 RNA polymerase system, which involves a recombinant vaccinia virus or fowlpox virus as a helper virus to produce T7 RNA polymerase (Peeters et al., 1999; Schnell et al., 1994). The helper viruses interfere with the efficiency of generating recombinant viruses from cDNA clones, especially for low-virulence strains. It also may lead to contamination of rescued virus, resulting in a significant amount of time to eliminate the helper virus. To overcome these deficiencies, Römer-Oberdörfer et al. (1999) utilized the BSR-T7/5 cell line, which could stably express the T7 RNA polymerase, to generate recombinant NDV from full-length genome cDNA clone. The system is simple and convenient, although it requires the maintenance of a special cell line to continuously express T7 RNA polymerase. In this study, we applied RNA polymerase II, a eukaryotic cell polymerase that is widely present in a variety of eukaryotic cells, which initiates the transcription of genome RNA from cDNA clones as well as mRNAs from three co-transfected helper plasmids that express the *NP*, *P*, and *L* proteins. The CMV promoter-controlled system is easier to operate and is efficient in rescuing recombinant viruses from cDNA clones (Li et al., 2011; Wang et al., 2015; Martin et al., 2006).

The naturally occurring lentogenic strain of NDV is widely used worldwide as a vaccine to control ND in the poultry industry and has a validated safety record, such as strains of LaSota and B1. NDV has a modular genome and can accommodate the insertion and expression of foreign genes. NDV is an optimal vector candidate that presents significant advantages compared to other viruses (Nakaya et al., 2001; Zhang et al., 2015; Kim and Samal, 2016). Recombinant NDV can efficiently express foreign proteins; therefore, it could be used as a vector to deliver protective antigens of other infectious agents to develop vector vaccines or to transfer genes for gene therapy purposes. Some isolates are naturally occurring oncolytic viruses. The reverse genetic system has the potential to enhance the targeted ability of recombinant viruses to cancer cells. Here, we constructed and rescued a recombinant NDV based on the Mukteswar strain expressing the EGFP gene, which was inserted between the non-coding region of *P* /*M* genes. The EGFP gene was stably expressed for at least 10 passages, including the 2 genetic markers.

Many efforts have been made to develop bivalent or multivalent vector vaccines based on recombinant NDV. There have been many attempts to insert protective antigens from various pathogens into the genome of NDV to study the immune efficacy of recombinant viruses as vector vaccines. These recombinant viruses induce different levels of immune responses due to the different insertion sites of foreign genes, the different lengths and expression levels of foreign genes, and whether foreign proteins can be integrated into recombinant viral particles. In addition, some infectious agents have more than one protective antigen, so the expression of only one antigen via recombinant NDV is not sufficient to produce complete immune protection. Therefore, some researchers have attempted to improve immune protection by inserting foreign genes as independent transcriptional units into different loci of the NDV genome, or by inserting a second exogenous gene in a second transcriptional reading frame based on the IRES sequence or a viral 2A self-cleaving peptides to express two exogenous genes (Ramp et al., 2011; Hu et al., 2017; Hu et al., 2018; He et al., 2020). However, gene expression of NDV is characterized by a decreasing gradient from the 3′ leader to the 5′ trailer of the genome. Therefore, there was a significant difference in the expression levels of the two exogenous genes using the above-mentioned methods, and results of clinical trials showed that different degrees of protection were obtained against the target pathogens. Although many factors affect the immune effectiveness of vector vaccines, there is no doubt that the expression efficiency of foreign genes is the most important factor. In order to achieve the simultaneous and efficient expression of two exogenous genes, we used the GE-GJ-GS motif of NDV as an adapter to link two foreign genes as two independent transcription units and inserted them into the optimal site of the NDV genome and rescued the recombinant virus. The results showed that the two exogenous genes could be expressed synchronously and efficiently, and the expression efficiency was even higher than that of the recombinant virus with a single exogenous gene. Thus, this method makes it possible to simultaneously express two or more foreign genes at the optimal insertion site of the NDV genome and is easier to manipulate. Therefore, this approach has the potential to develop bivalent or multivalent vector vaccines with better immune efficacy to reduce the cost of vaccination and avoid interference phenomena between different strains of vaccines. Theoretically, this method can also be applied to other mononegaviruses. The quantitative measurement of fluorescence showed that the expression level of CFP was significantly higher than EGFP. As described above, NDV is characterized by a gradient decrease of gene expression from the 3′leader to the 5′trailer. The two genes of EGFP and CFP are inserted sequentially, so theoretically, the expression levels between the two should be different. Why is the intensity of CFP higher than that of EGFP? Conversely, confocal photographs show that EGFP appears to fluoresce more than CFP. This is an interesting phenomenon in the results of this study. This is different from the results of a study conducted by He et al. (2020) using the LaSota strain as a vector. It is also different from the long-held belief that the gene expression of NDV has the characteristic of a gradient decrease from the 3′ end to the 5′ end of the genome. What causes such an outcome? Further research is needed. In addition, in the case of these two genes, the gene length is not long, and studies have shown that the length of exogenous genes is also an important factor affecting expression levels. In future studies, the length of exogenous genes should be fully considered to weigh their position in the genome and achieve the ideal expression level at the same time.

Some studies have concluded that with the increase in the number of genes in the NDV genome, the expression efficiency of the whole genome will be significantly affected. However, in this study, the results of quantitative detection showed that there was no significant difference in the expression efficiency of EGFP between rMuk-EGFP and rMuk-E/C. Why this phenomenon occurs, we suppose the reason may be that Mukteswar is a mesogenic strain, and it can cause more severe damage to cultured cells than lentogenic strains, as evidenced by the fact that it forms very significant syncytia in cultured cells. Although the insertion of double exogenous genes did not significantly change the virulence of the recombinant virus, this can be reflected in the MDT value. It also suggests that the rMuk-E/C-infected cells may have a slower pathological process compared to rMuk-EGFP-infected cells, resulting in the gene expression efficiency of rMuk-E/C being higher than that of rMuk-EGFP.

In summary, we established a reverse genetic system of the mesogenic NDV strain, which is controlled by the CMV promoter. Based on this reverse genetic system, we developed a method to simultaneously express two foreign genes at the optimal insertion site as two independent transcription units. The results showed that both exogenous genes could be expressed efficiently, and there was no significant difference in the expression efficiency of EGFP compared with recombinant virus rMuk-EGFP. A phenomenon of concern is that the expression efficiency of the second exogenous gene, CFP, is significantly higher than that of EGFP. Whether this will happen if two genes for other pathogens are co-expressed in this way remains to be confirmed by further studies. The virulence of two recombinant viruses was slightly attenuated compared to the parent virus. NDV is an ideal vaccine vector candidate for disease prevention in humans and animals. The modularity of the NDV genome, the low probability of recombination, the fact that the entire replication process takes place in the cytoplasm, and the absence of the DNA stage make NDV an advantageous candidate for the development of multiple applications in the biomedical field. Research in the field of cancer therapy has shown that NDV can infect and lyse tumor cells without damaging normal cells and can activate the body’s anti-tumor immune response in multiple pathways (Tayeb et al., 2015). More importantly, the absence of antibodies specific to NDV in the general population makes it extremely attractive to develop vector vaccines and oncolytic therapeutics. In a word, this is a novel method that uses the GE-GJ-GS motif as a linker to enable the simultaneous and efficient expression of two foreign genes as two independent transcription units in the NDV genome, which has the potential to develop multivalent vector vaccines, improve the immune protective effect of bivalent vector vaccines, or for tumor therapy purposes. It may also be used in other mononegaviruses.

## Data Availability

The datasets generated for this study are available on request to the corresponding author.
